# Gender Differences in Guideline-Directed Medical Therapy for Cardiovascular Disease Among Young Veterans

**DOI:** 10.1007/s11606-022-07595-1

**Published:** 2022-08-30

**Authors:** Sanket S. Dhruva, James Dziura, Harini Bathulapalli, Lindsey Rosman, Allison E. Gaffey, Melinda B. Davis, Cynthia A. Brandt, Sally G. Haskell

**Affiliations:** 1 San Francisco Veterans Affairs Health Care System, San Francisco, CA USA; 2 Section of Cardiology, Department of Medicine, UCSF School of Medicine, 4150 Clement St., Building 203, 111C, San Francisco, CA 94121 USA; 3 Veterans Affairs Connecticut Healthcare System, West Haven, West Haven, USA; 4 Pain Research, Informatics, Multimorbidities and Education (PRIME) Center of Innovation, VA Connecticut Healthcare System, West Haven, Department of Emergency Medicine, Yale School of Medicine, New Haven, CT USA; 5 Department of Internal Medicine, Yale School of Medicine, New Haven, CT USA; 6 Department of Medicine, Division of Cardiology, University of North Carolina School of Medicine, Chapel Hill, NC USA; 7 Department of Internal Medicine (Cardiovascular Medicine), Yale School of Medicine, New Haven, CT USA; 8 Veterans Affairs Ann Arbor Health System, Ann Arbor, MI USA; 9 Department of Internal Medicine, Division of Cardiovascular Medicine, University of Michigan, Ann Arbor, MI USA; 10 Department of Obstetrics and Gynecology, University of Michigan, Ann Arbor, MI USA; 11 Department of Internal Medicine (General), Yale School of Medicine, New Haven, CT USA

**Keywords:** gender differences, cardiovascular disease, medical therapy, coronary artery disease, heart failure

## Abstract

**Background:**

There is an increasing burden of cardiovascular disease, including coronary artery disease (CAD) and heart failure (HF), among women Veterans. Clinical practice guidelines recommend multiple pharmacotherapies that can reduce risk of mortality and adverse cardiovascular outcomes.

**Objective:**

To determine if there are disparities in the use of guideline-directed medical therapy by gender among Veterans with incident CAD and HF.

**Design:**

Retrospective.

**Participants:**

Veterans (934,504; 87.8% men and 129,469; 12.2% women) returning from Operations Enduring Freedom, Iraqi Freedom, and New Dawn.

**Main Measures:**

Differences by gender in the prescription of Class 1, Level of Evidence A guideline-directed medical therapy among patients who developed incident CAD and HF at 30 days, 90 days, and 12 months after diagnosis. For CAD, medications included statins and antiplatelet therapy. For HF, medications included beta-blockers and renin-angiotensin-aldosterone system inhibitors.

**Key Results:**

Overall, women developed CAD and HF at a younger average age than men (mean 45.8 vs. 47.7 years, *p*<0.001; and 43.7 vs. 45.4 years, *p*<0.02, respectively). In the 12 months following a diagnosis of incident CAD, the odds of a woman receiving a prescription for at least one CAD drug was 0.85 (95% confidence interval [CI], 0.68–1.08) compared to men. In the 12 months following a diagnosis of incident HF, the odds of a woman receiving at least one HF medication was 0.54 (95% CI, 0.37–0.79) compared to men.

**Conclusions:**

Despite guideline recommendations, young women Veterans have approximately half the odds of being prescribed guideline-directed medical therapy within 1-year after a diagnosis of HF. These results highlight the need to develop targeted strategies to minimize gender disparities in CVD care to prevent adverse outcomes in this young and growing population.

## INTRODUCTION

Cardiovascular disease (CVD) is a leading cause of disability, hospitalization, and premature death among women in the USA.^1,2^ CVD risk factors are common among women Veterans and are increasing in women at younger ages.^3^ The number of women Veterans has been growing more rapidly than men, with the number of women Veterans projected to increase 0.6% per year, compared to an annual decrease of 2.2% per year for men through 2045;^4^ more than half of women Veterans returning from the Iraq and Afghanistan conflicts have enrolled in care from the Department of Veterans Affairs (VA). Therefore, CVD management for women Veterans is increasingly important in order to reduce morbidity and mortality.

American College of Cardiology/American Heart Association (ACC/AHA) clinical practice guidelines provide recommendations and standards of care for treatment of patients with CVD, including stable ischemic heart disease/coronary artery disease (CAD) and heart failure (HF).^5–9^ The guidelines recommend, with a high level of evidence, several medical therapies that have been shown to reduce adverse cardiovascular outcomes in randomized, controlled trials. However, data from non-Veteran populations have shown that many patients do not receive guideline-directed medications,^10,11^ and women are often less likely to receive these therapies compared to men.^12,13^

Within the population of patients receiving care within VA, more than 10% of women Veterans have at least one established CVD condition,^14^ underscoring the importance of determining if gender-based disparities observed in the general population are also present among Veterans with CVD. Gender-based inequalities in the VA system were initially noted in 2006 when the VA began examining performance measure data for management of multiple conditions, such as control of low-density lipoprotein (LDL) cholesterol among Veterans with ischemic heart disease, by gender.^15^ Fortunately, these disparities have narrowed for most—but not all—cardiovascular risk factors.^15^ Although prior research has examined risk factors, only one study has examined possible disparities by gender in established CVD.^16^ Furthermore, no research has examined if there are gender differences in medical therapy for HF among Veterans. Given the increasing burden of CVD in women Veterans and limited data on guideline-directed medical therapy (GDMT) in this high-risk population, we sought to determine if there are gender differences in the provision of outpatient GDMT for CAD and HF. We focused on Veterans returning from recent Operations Enduring Freedom, Iraqi Freedom, and New Dawn (OEF/OIF/OND). As these are younger patients than the general Veteran population, and given the lifelong burden of CVD, understanding possible gender-based disparities is particularly important as GDMT can reduce the risk of later adverse outcomes.

## METHODS

### Data Source

The study cohort was formed using the Department of Defense OEF/OIF/OND roster of Veterans who participated in military service during conflicts in Iraq and Afghanistan and enrolled for VA care. This dataset is maintained by the Defense Manpower Data Center–Contingency Tracking System Deployment File, which contains sociodemographic and military service data for all men and women who were discharged from the US military and enrolled in VA care from October 1, 2001 (the start of US operations in Afghanistan), through October 1, 2015. This is a population of 1,063,973 individuals, including 934,504 (87.8%) men and 129,469 (12.2%) women.

All Veterans from the roster were included in the Women Veterans’ Cohort Study, an investigation of gender-based disparities in women’s health, risk factors, and healthcare utilization.^17,18^ To create the study dataset, individual’s demographic data were linked with VA inpatient, outpatient, pharmacy, and medical claims data from the VA Corporate Data Warehouse. Medical claims data for non-VA care provided to Veterans were also obtained from the VA Fee Basis Inpatient and Outpatient data. Clinical diagnoses were considered incident if a new (i.e., the first occurrence) International Classification of Diseases, Ninth Revision-Clinical Modification (ICD-9-CM) code for a specific condition was recorded during a hospitalization or a minimum of two outpatient encounters. This method is commonly used for research conducted with VA and administrative claims data and has demonstrated enhanced accuracy of diagnosis identification.^19^ Study baseline was defined as the date of a patient's first VA clinical encounter on or after October 1, 2001, and patients with any history of CAD or HF at their first VHA encounter (baseline) were excluded. The project and waiver of informed consent were approved by the VA Connecticut Healthcare System (West Haven, CT) Institutional Review Board.

### Coronary Artery Disease and Heart Failure Diagnoses

As in prior studies, CAD cases were ascertained using automated computer algorithms and validated ICD-9-CM codes (410.X [acute myocardial infarction], 411.X [other acute and subacute forms of ischemic heart disease], 414.X [other forms of chronic ischemic heart disease], 429.7 [certain sequelae of myocardial infarction not elsewhere classified], V45.81 [prior coronary artery bypass graft surgery] and V45.82 [prior percutaneous coronary intervention]).^20,21^ Similar methods were used to identify cases of HF (402.X [hypertensive heart disease, using only codes with accompanying heart failure], 404.X [hypertensive heart and chronic kidney disease], and 428.X [heart failure]).^22^ To ensure the identification of patients with reduced left ventricular systolic function, those patients with only diastolic HF codes (428.3X) were excluded. Furthermore, because some guideline-directed medications (e.g., angiotensin converting enzyme [ACE] inhibitors or angiotensin receptor blockers [ARB]) are contraindicated in pregnant patients,^23^ we excluded cardiac events that occurred 1 year before and after any pregnancy-related diagnosis.

### Covariates

Covariates were defined a priori and included demographics, cardiovascular risk factors, and other conditions that could potentially have a relationship with GDMT for CAD and HF. Demographic covariates were age, race, military rank, and marital status. Traditional cardiovascular risk factors were smoking, diabetes, hypertension, and dyslipidemia. Other conditions included were alcohol use, kidney disease, pulmonary disease, posttraumatic stress disorder (PTSD), and major depression. We also examined the association of primary care visits with GDMT.

### Medication Information

GDMT for CAD and HF with reduced ejection fraction (HFrEF) were identified as therapies with a Class 1, Level of Evidence A recommendation using ACC/AHA clinical practice guidelines.^5–9^ Class 1 recommendations are those for which the benefits significantly outweigh risks, and the treatment should be administered. Level of Evidence A means that data supporting the recommendation are derived from multiple randomized clinical trials or meta-analyses. For CAD, patient receipt of 1) either aspirin or clopidogrel and 2) a high-intensity statin was included. We also included beta-blocker prescriptions, since this class of medications is recommended (Class 1) for patients with an acute coronary syndrome, which is often the presenting marker of CAD, and these medications are also frequently used in CAD. As there are published data on underuse of statins and some patients are started at low or moderate doses, we also sought to examine if any cholesterol-lowering therapy was prescribed (including bile-acid sequestrants, fibrates, omega-3 fatty acids, and any statin dose). For HF, patient receipt of both a composite of the following medication classes and each class independently, 1) guideline-recommended beta-blocker (i.e., metoprolol [we examined any metoprolol and not just metoprolol succinate, since patients are often started and titrated on metoprolol tartrate and then transitioned to metoprolol succinate], carvedilol, or bisoprolol); 2) ACE inhibitor or ARB; and 3) mineralocorticoid receptor antagonist was included.

### Statistical Analysis

Bivariate descriptive statistics were first used to compare the characteristics of men and women with CAD and HF, using chi-square tests for categorical variables and *t*-tests for continuous variables. Multivariable logistic regression was then performed to identify independent variables (gender, demographics, cardiovascular risk factors, additional relevant conditions, and primary care visits) that were associated with the use of at least one guideline-directed medication for CAD (aspirin, clopidogrel, beta-blocker, cholesterol-lowering therapy, and the subset of high-intensity statins) by gender at 1 year. We also performed an identical multivariable logistic regression to compare the prescription of a new guideline-directed medication (i.e., one that the patient had not been prescribed before the incident CAD diagnosis, to account for the fact that some patients may already have been prescribed one of the medications for a different indication) by gender at 1 year. Analyses were first conducted to examine unadjusted odds ratios. Next, three sequential models were constructed. In model 1, covariates included demographic variables (age, race/ethnicity, rank, education, marital status), smoking, alcohol use, pulmonary disease, select traditional cardiovascular risk factors (diabetes and kidney disease), and non-traditional cardiovascular risk factors that are common in this patient population^14^ (major depression and PTSD). In model 2, two additional cardiovascular risk factors were added—hypertension and dyslipidemia—because the medications used to treat these conditions include beta-blockers, ACE inhibitors, and ARBs for hypertension and cholesterol-lowering medications for dyslipidemia. In model 3, an indicator of the number of primary care visits within 2 years after incident diagnosis was added to determine the potential effect of interactions with primary care clinicians, which would provide an opportunity for medication prescription. Finally, analogous multivariable logistic regression models were created to determine how gender is associated with at least one guideline-directed medication for HF (ACE inhibitor, ARB, guideline-recommended beta-blocker [bisoprolol, carvedilol, or metoprolol], and/or mineralocorticoid receptor antagonist). As the rate of missing data was low for covariates in models 1 and 2 (i.e., <1.1% overall for both CAD and HF analyses), a listwise deletion was used excluding individuals with any missing data in multivariable analyses. Although the number of primary care visits was missing more often, a complete case analysis was still used in model 3 as we were only investigating its impact on the odds ratios comparing women to men. Overall, among patients with an incident CAD diagnosis, 74/486 (15.2%) women and 1182/9285 (12.7%) men had missing data; this was not significantly different (*p*=0.11). Among patients with incident HF diagnoses, 20/188 (10.6%) women, 245/1970 (12.4%) men had missing data; this was also not significantly different (*p*=0.47). All analyses were conducted using SAS version 9.4 (SAS Institute, Inc., Cary, NC).

## RESULTS

### Characteristics of Patients with Coronary Artery Disease

Overall, 9771 patients developed CAD, 5.0% of whom were women (Table 1). Compared to men, women were significantly younger when they were diagnosed with CAD (mean age 45.8 vs. 47.7 years, *p*<0.001). A higher percentage of women who developed CAD were Black and were more likely to have completed at least a high school education than men. Women were less likely to have a diagnosis of alcohol abuse or to smoke compared with men. Women were less likely to have hypertension and dyslipidemia than men. There was no statistically significant difference in the percentage of men and women with a diagnosis of PTSD, but women were more likely to have a diagnosis of major depressive disorder than men. Finally, women had lower rates of prescription of cholesterol-lowering medication, clopidogrel, and beta-blockers in the 90 days prior to CAD diagnosis.

**Table 1:** Demographics for Veterans with a Diagnosis of Coronary Artery Disease by Gender and Overall

	Women (*N* = 486)	Men (*N* = 9285)	Total (*N* = 9771)	*p* value
Age^				
Mean (SD)	45.8 (9.6)	47.7 (9.8)	47.6 (9.8)	<0.001***
Race and ethnicity				<0.001***
Black	193 (39.7%)	1716 (18.5%)	1909 (19.5%)	
Hispanic	44 (9.1%)	1044 (11.2%)	1088 (11.3%)	
Other or unknown	35 (7.2%)	712 (7.7%)	747 (7.6%)	
White	214 (44.0%)	5813 (62.6%)	6027 (61.7%)	
Military rank				<0.004**
Enlisted	417 (85.8%)	8223 (88.6%)	8640 (88.4%)	
Officer	65 (13.4%)	879 (9.5%)	944 (9.7%)	
Warrant	4 (0.8%)	183 (2.0%)	187 (1.9%)	
Branch of service				<0.001***
Army	325 (66.8%)	6347 (68.4%)	6672 (68.3%)	
Coast Guard	0 (0%)	17 (0.2%)	17 (0.2%)	
Air Force	87 (17.9%)	1454 (15.7%)	1541 (15.8%)	
Marines	3 (0.6%)	404 (4.4%)	407 (4.2%)	
Navy	71 (14.6%)	1063 (11.4%)	1134 (11.6%)	
Highest education completed^				<0.001***
Less than high school	2 (0.4%)	102 (1.1%)	104 (1.1%)	
High school	277 (57.0%)	6135 (66.1%)	6412 (65.6%)	
More than high school	207 (42.6%)	3046 (32.8%)	3253 (33.3%)	
Marital status^				<0.001***
Married	237 (48.8%)	6847 (73.7%)	7084 (72.5%)	
Not married	248 (51.0%)	2434 (26.2%)	2682 (27.4%)	
Smoking status^				<0.001***
Never	244 (50.9%)	3415 (37.2%)	3659 (37.4%)	
Current smoker	177 (37.0%)	4124 (44.9%)	4301 (44.0%)	
Former smoker	58 (12.1%)	1650 (18.0%)	1708 (17.5%)	
Clinical variables				
Diabetes mellitus	101 (20.8%)	2351 (25.3%)	2452 (25.1%)	0.02*
Hypertension	288 (59.3%)	6561 (70.7%)	6849 (70.1%)	<0.001***
Alcohol abuse	62 (12.8%)	1676 (18.1%)	1738 (17.8%)	0.003**
Dyslipidemia	298 (61.3%)	7386 (79.6%)	7684 (78.6%)	<0.001***
Peripheral arterial disease	23 (4.7%)	442 (4.8%)	465 (4.8%)	0.98
Chronic kidney disease	20 (4.1%)	477 (5.1%)	497 (5.1%)	0.32
Pulmonary disease	154 (31.7%)	1563 (16.8%)	1717 (17.6%)	<0.001***
Post-traumatic stress disorder	239 (49.2%)	4330 (46.6%)	4569 (46.8%)	0.27
Major depressive disorder	198 (40.7%)	2034 (21.9%)	2232 (22.8%)	<0.001***
Transient ischemic attack	9 (1.9%)	134 (1.4%)	143 (1.5%)	0.46
Number of primary care visits in 2 years after diagnosis				
Mean (SD)	7.7 (5.9)	6.3 (5.9)	6.4 (5.9)	<0.001***
Medication prescriptions within 90 days prior to CAD diagnosis				
Cholesterol-lowering medication	110 (22.6%)	2797 (30.1%)	2907 (29.8%)	<0.001***
Aspirin	58 (11.9%)	1403 (15.1%)	1461 (15.0%)	0.06
Clopidogrel	19 (3.9%)	663 (7.1%)	682 (7.0%)	0.006**
Beta-blocker	90 (18.5%)	2148 (23.1%)	2238 (22.9%)	0.02^*^

* *p* value less than 0.05

**p value less than 0.01

***p value less than 0.001

*CAD*, coronary artery disease

^Unknown values: age (1 man), highest education completed (2 men), marital status (1 woman and 4 men), and smoking status (7 women and 96 men)

### Guideline-Directed Medical Therapy by Gender After Coronary Artery Disease Diagnosis

There were significant differences by gender in the prescription of GDMT following a diagnosis of CAD (Table 2). Of the 486 women and 9285 men who were diagnosed with CAD, 34.2% of women were prescribed any of either aspirin, clopidogrel, beta-blocker, or cholesterol-lowering therapy within 30 days, compared to 43.9% of men (*p*<0.001). Within 90 days of diagnosis, 51.9% of women vs. 60.4% of men had been prescribed at least one of any of these medications (*p*<0.001). There was a similar gender difference at 1 year, which was notable across different GDMT. Approximately 11.8% of women were prescribed a high-intensity statin compared to 16.0% of men (*p*=0.01). Moreover, 40.5% of women had been prescribed a beta-blocker compared to 48.9% of men (*p*<0.001). These gender differences also persisted across most prescriptions of new medications.

**Table 2:** Guideline-Directed Medical Therapy for Coronary Artery Disease by Gender and Overall

	Women (*N* = 486)	Men (*N* = 9285)	Total (*N* = 9771)	*p* value
Guideline-directed medical therapy prescription within 12 months of diagnosis				
Any medication for CAD	309 (63.6%)	6631 (71.4%)	6940 (71.0%)	<0.001***
Any new medication for CAD	209 (43.0%)	4612 (49.7%)	4821 (49.3%)	0.004**
Any cholesterol-lowering medication	226 (46.5%)	5682 (61.2%)	5908 (60.5%)	<0.001***
Any new cholesterol-lowering medication	120 (24.7%)	3041 (32.8%)	3161 (32.4%)	<0.001***
Any high-intensity statin	57 (11.7%)	1490 (16.0%)	1547 (15.8%)	0.011**
Any new high-intensity statin	37 (7.6%)	758 (8.2%)	795 (8.1%)	0.67
Aspirin	132 (27.2%)	3278 (35.3%)	3410 (34.9%)	<0.001***
New aspirin	88 (18.1%)	2095 (22.6%)	2183 (22.3%)	0.021*
Clopidogrel	58 (11.9%)	1826 (19.7%)	1884 (19.3%)	<0.001***
New clopidogrel	41 (8.4%)	1265 (13.6%)	1306 (13.4%)	<0.001***
Beta-blocker	197 (40.5%)	4542 (48.9%)	4739 (48.5%)	<0.001***
New beta-blocker	119 (24.5%)	2568 (27.7%)	2687 (27.5%)	0.13
Guideline-directed medical therapy prescription within 90 days of diagnosis				
Any medication for CAD	252 (51.9%)	5612 (60.4%)	5864 (60.0%)	<0.001***
Any new medication for CAD	157 (32.3%)	3612 (38.9%)	3769 (38.6%)	0.004**
Any cholesterol-lowering medication	174 (35.8%)	4417 (47.6%)	4591 (47.0%)	<0.001***
Any new cholesterol-lowering medication	92 (18.9%)	2248 (24.2%)	2340 (23.9%)	0.008**
Aspirin	95 (19.5%)	2343 (25.2%)	2438 (25.0%)	0.005**
New aspirin	61 (12.6%)	1506 (16.2%)	1567 (16.0%)	0.03*
Clopidogrel	51 (10.5%)	1539 (16.6%)	1590 (16.3%)	<0.001***
New clopidogrel	35 (7.2%)	1062 (11.4%)	1097 (11.2%)	0.004**
Beta-blocker	146 (30.0%)	3588 (38.6%)	3734 (38.2%)	<0.001***
New beta-blocker	85 (17.5%)	1990 (21.4%)	2075 (21.2%)	0.04*
Guideline-directed medical therapy prescription within 30 days of diagnosis				
Any medication for CAD	166 (34.2%)	4072 (43.9%)	4238 (43.4%)	<0.001***
Any new medication for CAD	108 (22.2%)	2814 (30.3%)	2922 (29.9%)	<0.001***
Any cholesterol-lowering medication	100 (20.6%)	2835 (30.5%)	2935 (30.0%)	<0.001***
Any new cholesterol-lowering medication	59 (12.1%)	1721 (18.5%)	1780 (18.2%)	<0.001***
Aspirin	54 (11.1%)	1487 (16.0%)	1541 (15.8%)	0.004**
New aspirin	43 (08.8%)	1149 (12.4%)	1192 (12.2%)	0.02*
Clopidogrel	36 (07.4%)	1052 (11.3%)	1088 (11.1%)	0.007**
New clopidogrel	30 (06.2%)	835 (09.0%)	865 (08.9%)	0.03*
Beta-blocker	88 (18.1%)	2245 (24.2%)	2333 (23.9%)	0.002**
New beta-blocker	56 (11.5%)	1473 (15.9%)	1529 (15.6%)	0.010*

*p value less than 0.05

**p value less than 0.01

***p value less than 0.001

*CAD*, coronary artery disease

In the fully adjusted model at 1 year, the odds ratio for a woman with an incident diagnosis of CAD receiving at least one CAD medication was 0.85 (95% confidence interval [CI], 0.68–1.08) compared to men (Table 3). Similarly, the odds ratio for a woman receiving a new prescription for at least one CAD medication was 0.92 (95% CI, 0.74–1.13) compared to men.

**Table 3:** Multivariable Logistic Regression of Gender Differences in Guideline-Directed Medications Prescribed Among Patients Within 1 Year After Incident Coronary Artery Disease

Outcome	Odds ratio (95% confidence interval)	*p* value
Any medication (unadjusted)	0.71 (0.58 – 0.86)	<0.001
Any medication (adjusted*)	0.75 (0.61 – 0.92)	0.006
Any medication (adjusted**)	0.96 (0.78 – 1.20)	0.75
Any medication (adjusted***)	0.85 (0.68 – 1.08)	0.19
Any new medication (unadjusted)	0.77 (0.64 – 0.93)	0.006
Any new medication (adjusted*)	0.81 (0.67 – 0.98)	0.03
Any new medication (adjusted**)	0.93 (0.76 – 1.13)	0.46
Any new medication (adjusted***)	0.92 (0.74 – 1.13)	0.42

Men as the referent. *CAD*, coronary artery disease

*Model 1: Adjusted for age, race/ethnicity, rank, education, marital status, smoking, alcohol use, pulmonary disease, diabetes, kidney disease, major depression, and post-traumatic stress disorder (*n*=9665)

**Model 2: Adjusted for all variables in model 1 as well as hypertension and dyslipidemia (*n*=9665)

***Model 3: Adjusted for all variables in model 2 as well as for the number of primary care visits (*n*= 8514)

### Characteristics of Patients with Heart Failure

Overall, 2158 patients developed HF, 188 (8.7%) of whom were women (Table 4). Compared to men, women were younger when they were diagnosed with HF (mean age 43.7 vs. 45.4 years, *p*<0.02). As observed with CAD, women who developed HF were also more likely to be Black and to have completed at least a high school education than men. Women were less likely to consume alcohol and to have a diagnosis of hypertension, dyslipidemia, or chronic kidney disease compared to men. However, women with HF were more likely to have a diagnosis of depression than men.

**Table 4:** Demographics for Veterans with a Diagnosis of Heart Failure by Gender and Overall

	Women (*N* = 188)	Men (*N* = 1970)	Total (*N* = 2158)	*p* value
Age				
Mean (SD)	43.7 (9.7)	45.4 (11.0)	45.3 (11.0)	0.02**
Race and ethnicity				<0.001***
Black	81 (43.1%)	583 (29.6%)	664 (30.8%)	
Hispanic	17 (9.0%)	181 (9.2%)	198 (9.2%)	
Other or unknown	15 (8.0%)	110 (5.6%)	123 (5.7%)	
White	75 (39.9%)	1096 (55.6%)	1171 (54.3%)	
Military rank				0.31
Enlisted	180 (95.7%)	1852 (94.0%)	2032 (94.2%)	
Officer	8 (4.3%)	95 (4.8%)	103 (4.8%)	
Warrant	0 (0%)	23 (1.2%)	23 (1.1%)	
Branch of service				0.04*
Army	130 (69.1%)	1329 (67.5%)	1459 (67.6%)	
Coast Guard	0 (0%)	2 (0.1%)	2 (0.1%)	
Air Force	27 (14.4%)	245 (12.4%)	272 (12.6%)	
Marines	3 (1.6%)	129 (6.5%)	132 (6.1%)	
Navy	28 (14.9%)	265 (13.5%)	293 (13.6%)	
Highest education completed				0.02*
Less than high school	1 (0.5%)	34 (1.7%)	35 (1.6%)	
High school	128 (68.1%)	1486 (75.4%)	1614 (74.8%)	
More than high school	59 (31.4%)	450 (22.8%)	509 (23.6%)	
Marital status^				<0.001***
Married	70 (37.2%)	1274 (64.7%)	1344 (62.3%)	
Not married	118 (62.8%)	695 (35.3%)	813 (37.7%)	
Smoking status^				0.26
Never	69 (37.1%)	649 (33.2%)	718 (33.3%)	
Current smoker	93 (50.0%)	968 (49.5%)	1061 (49.2%)	
Former smoker	24 (12.9%)	338 (17.3%)	362 (16.8%)	
Clinical variables				
Diabetes mellitus	32 (17.0%)	496 (25.2%)	528 (24.5%)	0.01**
Hypertension	100 (53.2%)	1371 (69.6%)	1471 (68.2%)	<0.001***
Dyslipidemia	86 (45.7%)	1275 (64.7%)	1361 (63.1%)	<0.001***
Transient ischemic attack	1 (0.5%)	35 (1.8%)	36 (1.7%)	0.36
Peripheral arterial disease	184 (97.9%)	1860 (94.4%)	2044 (94.7%)	0.04**
Chronic kidney disease	18 (9.6%)	338 (17.2%)	356 (16.5%)	0.007**
Pulmonary disease	93 (49.5%)	753 (38.2%)	846 (39.2%)	0.003**
Post-traumatic stress disorder	97 (51.6%)	964 (48.9%)	1061 (49.2%)	0.49
Major depressive disorder	70 (37.2%)	462 (23.5%)	532 (24.7%)	<0.001***
Alcohol abuse	35 (18.6%)	522 (26.5%)	557 (25.8%)	0.02^*^
Number of primary care visits in 2 years after diagnosis				
Mean (SD)	7.1 (5.8)	7.1 (7.0)	7.1 (6.9)	0.99
Medication prescriptions within 90 days prior to HF diagnosis				
ACE inhibitor prescription	37 (19.7%)	536 (27.1%)	573 (26.6%)	0.03**
ARB prescription	6 (3.2%)	97 (4.9%)	103 (4.8%)	0.29
Bisoprolol prescription	0 (0%)	0 (0%)	0 (0%)	
Carvedilol prescription	17 (9.0%)	255 (12.9%)	272 (12.6%)	0.12
Metoprolol prescription	13 (6.9%)	303 (15.4%)	316 (14.6%)	0.002**
Mineralocorticoid receptor antagonist prescription	9 (4.8%)	103 (5.2%)	112 (5.2%)	0.79

*p value less than 0.05

**p value less than 0.01

***p value less than 0.001

*ACE*, angiotensin converting enzyme; *ARB*, angiotensin receptor blocker

^Unknown values: marital status (1 man), and smoking status (2 women and 15 men)

### Guideline-Directed Medical Therapy by Gender After Heart Failure Diagnosis

After a diagnosis of HF, there were significant differences by gender in the prescription of GDMT (Table 5). Of the 188 women and 1970 men, 21.8% of women were prescribed either an ACE inhibitor, ARB, guideline-recommended beta-blocker, or mineralocorticoid receptor antagonist within 30 days of diagnosis, compared to 37.3% of men (*p*<0.001). Within 90 days of diagnosis, 29.8% of women vs. 54.2% of men had been prescribed at least one of these medications (*p*<0.001). There was a similar difference at 1 year, which was notable across different guideline-recommended medications. Altogether, 31.9% of women were prescribed an ACE inhibitor or ARB, compared to 53.7% of men (*p*<0.001). Similarly, 30.9% of women were prescribed a guideline-recommended beta-blocker, compared to 49.5% of men (*p*<0.001). These differences also persisted across prescriptions of new medications.

**Table 5:** Guideline-Directed Medical Therapy for Heart Failure by Gender and Overall

	Women (*N* = 188)	Men (*N* = 1970)	Total (*N* = 2158)	*p* value
Guideline-directed medical therapy prescription within 12 months of diagnosis				
Any of ACE inhibitor, ARB, bisoprolol, carvedilol, metoprolol, or mineralocorticoid receptor antagonist	76 (40.4%)	1230 (62.4%)	1306 (60.5%)	<0.001***
Any new ACE inhibitor, ARB, bisoprolol, carvedilol, metoprolol, or mineralocorticoid receptor antagonist	43 (22.9%)	785 (39.8%)	828 (38.4%)	<0.001***
ACE inhibitor	49 (26.1%)	918 (46.6%)	967 (44.8%)	<0.001***
New ACE inhibitor	18 (9.6%)	435 (22.1%)	453 (21.0%)	<0.001***
ARB	17 (9.0%)	200 (10.2%)	217 (10.1%)	0.63
New ARB	12 (6.4%)	115 (5.84)	127 (5.9%)	0.76
Any ACE inhibitor or ARB	60 (31.9%)	1058 (53.7%)	1118 (51.8%)	<0.001***
Any new ACE inhibitor or ARB	27 (14.4%)	527 (26.8%)	554 (25.7%)	<0.001***
Bisoprolol	0 (0%)	3 (0.2%)	3 (0.1%)	1.00
New bisoprolol	0 (0%)	3 (0.2%)	3 (0.1%)	1.00
Carvedilol	35 (18.6%)	537 (27.3%)	572 (26.5%)	0.01**
New carvedilol	18 (9.6%)	306 (15.5%)	324 (15.0%)	0.03*
Metoprolol	27 (14.4%)	509 (25.8%)	536 (24.8%)	<0.001***
New metoprolol	16 (8.5%)	255 (12.9%)	271 (12.6%)	0.08
Bisoprolol, carvedilol, or metoprolol	58 (30.9%)	976 (49.5%)	1034 (47.9%)	<0.001***
New bisoprolol, carvedilol, or metoprolol	31 (16.5%)	530 (26.9%)	561 (26.0%)	0.002**
Mineralocorticoid receptor antagonist	20 (10.6%)	313 (15.9%)	333 (15.4%)	0.06
New mineralocorticoid receptor antagonist	12 (6.4%)	220 (11.2%)	232 (10.8%)	0.04*
Guideline-directed medical therapy prescription within 90 days of diagnosis				
Any of ACE inhibitor, ARB, bisoprolol, carvedilol, metoprolol, or mineralocorticoid receptor antagonist	56 (29.8%)	1068 (54.2%)	1124 (52.1%)	<0.001***
Any new ACE inhibitor, ARB, bisoprolol, carvedilol, metoprolol, or mineralocorticoid receptor antagonist	28 (14.9%)	605 (30.7%)	633 (29.3%)	<0.001***
ACE inhibitor	39 (20.7%)	742 (37.7%)	781 (36.2%)	<0.001***
New ACE inhibitor	16 (8.5%)	334 (17.0%)	350 (16.2%)	0.003**
ARB	10 (5.3%)	140 (7.1%)	150 (7.0%)	0.36
New ARB	7 (3.7%)	66 (3.4%)	73 (3.4%)	0.79
Any ACE inhibitor or ARB	46 (24.5%)	859 (43.6%)	905 (41.9%)	<0.001***
Any new ACE inhibitor or ARB	21 (11.2%)	394 (20.0%)	415 (19.2%)	0.003**
Bisoprolol	0 (0%)	2 (0.1%)	2 (0.1%)	1.00
New bisoprolol	0 (0%)	2 (0.1%)	2 (0.1%)	1.00
Carvedilol	27 (14.4%)	417 (21.2%)	444 (20.6%)	0.03**
New carvedilol	14 (7.4%)	227 (11.5%)	241 (11.2%)	0.09
Metoprolol	15 (8.0%)	401 (20.4%)	416 (19.3%)	<0.001***
New metoprolol	8 (4.3%)	188 (9.5%)	196 (9.1%)	0.02*
Bisoprolol, carvedilol, or metoprolol	41 (21.8%)	796 (40.4%)	837 (38.8%)	<0.001***
New bisoprolol, carvedilol, or metoprolol	21 (11.2%)	405 (20.6%)	426 (19.7%)	0.002**
Mineralocorticoid receptor antagonist	14 (7.4%)	227 (11.5%)	241 (11.2%)	0.09
New mineralocorticoid receptor antagonist	8 (4.3%)	146 (7.41%)	154 (7.1%)	0.11
Guideline-directed medical therapy prescription within 30 days of diagnosis				
Any of ACE inhibitor, ARB, bisoprolol, carvedilol, metoprolol, or mineralocorticoid receptor antagonist	41 (21.8%)	734 (37.3%)	775 (35.9%)	<0.001***
Any new ACE inhibitor, ARB, bisoprolol, carvedilol, metoprolol, or mineralocorticoid receptor antagonist	19 (10.1%)	422 (21.4%)	441 (20.4%)	<0.001***
ACE inhibitor	27 (14.4%)	455 (23.1%)	482 (22.3%)	0.006**
New ACE inhibitor	10 (5.3%)	233 (11.8%)	243 (11.3%)	0.007**
ARB	5 (2.7%)	74 (3.8%)	79 (3.7%)	0.44
New ARB	4 (2.1%)	37 (1.9%)	41 (1.9%)	0.78
Any ACE inhibitor or ARB	30 (16.0%)	525 (26.7%)	555 (25.7%)	0.001^**^
Any new ACE inhibitor or ARB	12 (6.4%)	269 (13.7%)	281 (13.0%)	0.005^**^
Bisoprolol	0 (0%)	1 (0.1%)	1 (0.0%)	1.00
New bisoprolol	0 (0%)	1 (0.1%)	1 (0.0%)	1.00
Carvedilol	21 (11.2%)	273 (13.9%)	294 (13.6%)	0.30
New carvedilol	10 (5.3%)	171 (8.7%)	181 (8.4%)	0.11
Metoprolol	8 (4.3%)	248 (12.6%)	256 (11.9%)	<0.001***
New metoprolol	5 (2.7%)	123 (6.2%)	128 (5.9%)	0.047*
Bisoprolol, carvedilol, or metoprolol	29 (15.4%)	513 (26.0%)	542 (25.1%)	0.001**
New bisoprolol, carvedilol, or metoprolol	15 (8.0%)	291 (14.8%)	306 (14.2%)	0.01*
Mineralocorticoid receptor antagonist	7 (3.7%)	130 (6.6%)	137 (6.3%)	0.12
New mineralocorticoid receptor antagonist	5 (2.7%)	92 (4.7%)	97 (4.5%)	0.20

*p value less than 0.05

**p value less than 0.01

***p value less than 0.001

*ACE*, angiotensin converting enzyme; *ARB*, angiotensin receptor blocker

In the fully adjusted model at 1 year, the odds ratio for a woman with an incident diagnosis of HF receiving at least one HF medication was 0.54 (95% CI, 0.37–0.79) compared to men (Table 6). Similarly, the odds ratio for a woman receiving a new prescription for at least one HF medication was 0.54 (95% CI, 0.36–0.80).

**Table 6:** Multivariable Logistic Regression of Gender Differences in Guideline-Directed Medications Prescribed Among Patients Within 1 Year After Incident Heart Failure

Outcome	Odds ratio (95% confidence interval)	*p* value
Any medication (unadjusted)	0.41 (0.30 – 0.56)	<.001
Any medication (adjusted*)	0.42 (0.30 – 0.59)	<.001
Any medication (adjusted**)	0.51 (0.35 – 0.73)	<.001
Any medication (adjusted***)	0.54 (0.37 – 0.79)	<.001
Any new medication (unadjusted)	0.45 (0.32 – 0.64)	<.001
Any new medication (adjusted*)	0.46 (0.31 – 0.66)	<.001
Any new medication (adjusted**)	0.51 (0.35 – 0.74)	<.001
Any new medication (adjusted***)	0.54 (0.36 – 0.80)	0.002

Men as the referent. *HF*, heart failure

*Model 1: Adjusted for age, race/ethnicity, rank, education, marital status, smoking, alcohol use, pulmonary disease, diabetes, kidney disease, major depression, and post-traumatic stress disorder (*n*=2141)

**Model 2: Adjusted for all variables in model 1 as well as hypertension and dyslipidemia (*n*=2141)

***Model 3: Adjusted for all variables in model 2 as well as for the number of primary care visits (*n*=1893)

## Discussion

Among more than 1 million young Veterans, women were significantly less likely than men to receive GDMT for a new diagnosis of CAD or HF. Although in the fully adjusted model, women with incident CAD did not have lower odds of receiving GDMT, the absolute rates were consistently lower in women. These findings reveal concerning care gaps, which may be contributing to worse cardiovascular outcomes in women than men. Disparities in the provision of evidence-based therapies need to be addressed to support improved secondary prevention and cardiovascular outcomes for women. Paired with findings from non-VA populations, our results indicate multiple opportunities and critical next steps to address disparities in prescription of GDMT.

Our findings add to a growing literature that demonstrates concerning gender disparities in cardiovascular care and outcomes, and particularly those which affect younger women. In civilian populations, younger women have worse cardiovascular outcomes than age-matched men and older women.^24^ Most prior studies of GDMT, however, have been conducted in older adults, who have a higher prevalence and a longer duration of exposure to established risk factors for cardiovascular disease.^12,13^ Thus, findings may not generalize to a younger patient population like ours, in which patients who were diagnosed with CAD and HF were, on average, in their 40s. Because both CAD and HF are lifelong conditions and their impact on morbidity and mortality can be mitigated through medical therapies,^5,9^ ensuring GDMT is especially important in younger patients. A recent VA study found similar gender-based disparities in the use of antiplatelet medications and statins among women with premature atherosclerotic CVD compared to men.^16^ Our study underscores the critical need to understand reasons for the relative undertreatment of women with guideline-concordant care.

In the present investigation, women who were newly diagnosed with CAD had more primary care visits than men and there was no gender difference among women and men newly diagnosed with HF, which suggests that access to routine care or interactions with the healthcare system are unlikely to be the key drivers of disparities. Alternatively, women may be less likely to be referred to cardiologists for management of CAD and HF; cardiologist referral has been associated with better performance on quality-of-care indicators based on national guidelines or standards for care of patients with CAD and HF.^25^ However, multiple studies in civilian populations have also identified gender bias by physicians, including cardiologists, in use of cardiovascular tests and treatments,^26,27^ partially explained by misperceptions of incidence among pre-menopausal women.^28^ Women Veterans more often have suboptimal experiences in specialty care, and clinician communication and education need improvement; for example, women Veterans have reported feeling that specialty clinicians did not take their symptoms seriously or told them that their health conditions or symptoms were attributable to hormonal fluctuations.^29^ VA clinicians are less likely to order LDL-lowering medications for women Veterans compared to men^30^ and women Veterans also decline lipid-lowering therapy more often than men,^30^ which may be attributable to sub-optimal clinician-patient communication, precluding women from understanding their risk.^31^

Another reason for the undertreatment of women with evidence-based medications may be related to the underlying etiology and manifestation of cardiovascular disease. Women Veterans undergoing coronary angiography are less likely to have obstructive CAD.^32^ Women are more likely to have microvascular CAD,^33^ and to present with myocardial infarction with non-obstructive coronary arteries (MINOCA) than men.^34^ Although the comparative lack of obstructive CAD among women may explain lower rates of prescribing aspirin and statins,^35^ these prescriptions are associated with better clinical outcomes.^36^ Albeit rare, among young women, a cause of acute coronary syndrome is spontaneous coronary artery dissection (SCAD), which has different clinical characteristics than atherosclerotic CAD. Although there is a paucity of currently available evidence about therapeutic strategies, patients who survive SCAD are often recommended for antiplatelet therapy and, if they meet other guideline-based indications, statins.^37^ Women are also more likely to have HF with preserved ejection fraction (HFpEF) compared to men.^38^ There are currently no guideline-recommended medications specifically to treat HFpEF. However, we excluded codes specific to HFpEF and it would be unexpected for coding to differ between men and women.

### Implications of Findings

As noted in a 2016 American Heart Association Scientific Statement, a key first step in implementing better use of GDMT is to increase education for women and clinicians about cardiovascular disease and the crucial role of GDMT.^28,39^ This education and awareness must include acknowledgement of the gender bias that leads women to receive less guideline-directed care.^27^ Second, research is needed to improve cardiovascular prevention in young adults, particularly women, through the development of targeted screening programs, patient counseling on individual risks, and age-appropriate interventions that are tailored to women. Electronic health record-based clinical decision support, which would provide objective recommendations regardless of gender, may be another strategy to reduce disparities in care.^27^ There is also a need to increase the enrollment of women in trials of therapies for cardiovascular disease.^40^ Third, care from gender-concordant clinicians may improve outcomes, including cardiovascular mortality;^41,42^ this requires additional investment and commitment to training women cardiologists.^43^ Finally, women Veterans are more likely to experience non-traditional CVD risk factors, including chronic stress, depression, and inadequate social support, which can adversely affect cardiovascular health.^44^ Attention to these barriers, including a heightened focus on mental health and social determinants of health, will be crucial to improving outcomes.^45,46^

Our findings should be interpreted in the context of several limitations. First, administrative claims were used to identify patients with CAD and HF. While claims have been validated and used in multiple prior studies,^20,22^ it is possible that these diagnoses were ascertained incorrectly and could explain the relatively low rates of GDMT prescriptions identified. We focused on GDMT for HFrEF but, as noted above, some patients with a diagnosis of HF may not have had HFrEF and, thus, would not have met criteria to be prescribed GDMT. However, we removed any codes suggesting diastolic HF/HFpEF and we would not expect the administrative claims codes to be used differently between women and men. Second, ICD-9 diagnoses may not necessarily correlate with the clinical timing of diagnosis and could explain why some patients were already on GDMT prior to their diagnosis. However, there are also other clinical reasons why some GDMT may have been prescribed. Third, aspirin ascertainment was included among patients with CAD. Because some patients purchase aspirin over-the-counter, it was not possible to account for all aspirin use. However, it is not expected that there would be a gender-based difference in over-the-counter use of aspirin. Fourth, we did not include prasugrel and ticagrelor as antiplatelet medications. Both the guidelines for stable ischemic heart disease^5^ and prevention of cardiovascular disease in women^1^ specifically listed only clopidogrel as an alternative to aspirin; although prasugrel and ticagrelor may substitute for clopidogrel, these were only approved for a fraction of the study time period and were nearly always non-formulary drug options within the VA during that time. Fifth, the analyses only accounted for medications prescribed through VA and did not account for patients who received prescription medications outside of VA. We also did not exclude patients without follow-up within VA. Some patients could have received GDMT from non-VA clinicians and prior research has shown that a greater percentage of women receive care from both VA and non-VA clinicians (57% of women vs. 51% of men) and solely non-VA clinicians (17% of women vs. 14% of men).^18^ This is an area for future investigation. Finally, the present study only examined if specific medications were prescribed and not if the doses were optimized; further research is required to examine if patients reach goal doses of medications.^9^

In conclusion, women Veterans were less likely than men to receive GDMT at 30 days, 90 days, and 1 year after diagnosis of CAD or HF, with significantly lower odds among women compared to men with HF. These findings were consistent across a variety of well-established medications that have been shown to reduce cardiovascular risk and improve outcomes for men and women. Attending to these disparities in care is of urgent importance to prevent women from experiencing a disproportionate, life-long, and more severe burden of CVD.
